# A Literature Review to Assess Blood Loss in Minimally Invasive Liver Surgery Versus in Open Liver Resection

**DOI:** 10.7759/cureus.16008

**Published:** 2021-06-28

**Authors:** Eiad Elmahi, Yahya Salama, Fergal Cadden

**Affiliations:** 1 General Surgery, Lincoln County Hospital, Lincoln, GBR; 2 Surgery, Kettering General Hospital, Kettering, GBR

**Keywords:** “laparoscopic hepatectomy”, “post-operative complications”, “post-hepatectomy bleeding”, “intra-operative bleeding”, “open liver resection”, “hepatocellular carcinoma”, “benign liver tumours”, “minimally invasive”, “hepatobiliary surgery”, “segmentectomy”. the term and “versus” was used to identify studies which compared the outcomes of both techniques

## Abstract

Aim and objectives

The aim of the study was to assess the amount of blood loss in minimally invasive hepatectomy and open liver resection for both benign and neoplastic conditions.

Introduction

Minimally invasive surgery has progressively developed to a stage where once-novel and highly specialized surgical techniques are now common practice. Colorectal surgery is the key example that has shown minimally invasive surgery as highly beneficial. Successes in the colorectal laparoscopic approach have now been integrated into the speciality of hepatopancreaticobiiary (HPB) surgery. In this review, we will compare the amount of blood loss in minimally invasive liver resection with the more traditional approach of open liver resection.

Methods

A literature review was conducted which included the length of patient mobilization as a postoperative complication following laparoscopic and open liver resections. Medline, PubMed, and Cochrane were accessed to review previously published studies. Twelve studies were selected, and all of them were in English, ranged from the year 2000 to 2020.

Results

Eleven out of the 12 included studies indicated that minimally invasive liver resection is associated with reduced blood loss.

Conclusion

In comparing both minimally invasive liver resection and classic open surgery, minimally invasive liver resection was shown to have reduced blood loss; this was seen in both malignant and benign tumours. Therefore, laparoscopic liver resection could be favoured over the classical open approach to avoid excessive blood loss intra-operatively

## Introduction

The introduction of the laparoscopic approach to many surgical practices has greatly altered the standard practice of many surgical specialities, through repeated reviews in the literature, it has proven to be superior in terms of postoperative outcomes for patients. It has seen a slow adaptation to HPB surgery in general where an open approach is opted for more frequently. The laparoscopic approach is less commonly applied for liver resection. This has been a point of contention as there are few studies to objectively compare the outcomes of the laparoscopic technique to the open technique. However, with the few studies that directly compare both practices, there are indications that a laparoscopic approach show reduced rates of postoperative complications [1].

In this study, we attempt to collate the available evidence for laparoscopic liver resection (LLR) and show evidence that there are benefits to the practice of laparoscopic liver resection compared to open liver resection (OLR). Comparing the evidence on minimally invasive and open techniques in liver resection will provide further insight into the premise that the laparoscopic approach reduced the amount of blood loss.

Postoperative hospital stay is a longstanding issue regarding a patient's recovery; prolonged hospital stay is associated with an increased risk of developing hospital-acquired infections and at a greater cost to the hospital [2]. By reviewing the available evidence, we hope to see that minimally invasive surgery helps facilitate reduced postoperative hospital stay. If the evidence suggests that the laparoscopic approach reduces the amount of blood loss intra-operatively, minimally invasive surgery may be recommended for liver resection over the traditional approach of open liver resection [3].

Postoperative bleeding using both techniques will be weighed. The type of liver resection and the age group (45-70 years) will be considered in this review to avoid complications not related to hepatectomies (cardio-respiratory, anaesthesia-related complications) [4].

Liver tumours are classified as benign or malignant, they are not uncommon. The most common benign liver tumour is haemangioma, which mainly affects the right lobe of the liver. In general, haemangiomas are usually asymptomatic and are usually associated with certain conditions like Klippel-Trenaunary syndrome. These are usually found as incidental findings during imaging (ultrasound scan and CT) [5]. The indications for liver resections in these cases include right upper abdominal fullness, bleeding, hemorrhagic shock, and gastric outlet obstruction [6]. Other types of benign tumours are hepatic adenoma which is more frequently associated with females on the oral contraceptive pill [7]. Rare benign tumours include cystadenoma, lipoma, fibroma, and leiomyoma which are symptomatic unless they cause complications [5].

Malignant tumours of the liver are the sixth most common cause of death globally and the third cause of mortality. Liver malignancy is more common in Asia and Central Africa; this could be attributed to aflatoxin-contaminated peanuts intake which is associated with a higher incidence of developing primary liver malignancy [8].

In terms of liver malignancy, they are categorized into primary or secondary. Primary liver malignancy is either a hepatoma or hepatocellular carcinoma (HCC). A secondary malignancy is caused by liver metastasis. Risk factors for HCC are varied, including high BMI, male gender, liver cirrhosis, high alcohol consumption, aflatoxin-contaminated food, and hepatitis [8].

Liver metastasis is the most common indication for liver resection, and colorectal cancer is the commonest primary source [9]. Hepatectomy is offered to patients with localized disease; unfortunately, approximately half of the patients develop disease recurrence [9]. The reasoning behind liver resection in metastatic colorectal disease is that there is a potential for a curative outcome and a better prognosis with surgical intervention than conservative treatment [8].

The use of laparoscopic liver resection is an umbrella term to describe different techniques of liver resection. These techniques include pure laparoscopy, hand-assisted laparoscopy, and laparoscopy-assisted methods [10]; these subtypes will also be included in this review.

Liver resection is considered a major operation. Its complications of course will need to be anticipated as postoperative complications are likely to develop in both anatomical and non-anatomical liver resections. Complications include bleeding, infection, and injury to surrounding structures, bile leak, and increasing length of postoperative stay. As expected, these reflect increased risk in morbidity and mortality as well as a prolonged hospital stay [11].

## Materials and methods

Search strategy

**Figure 1:**
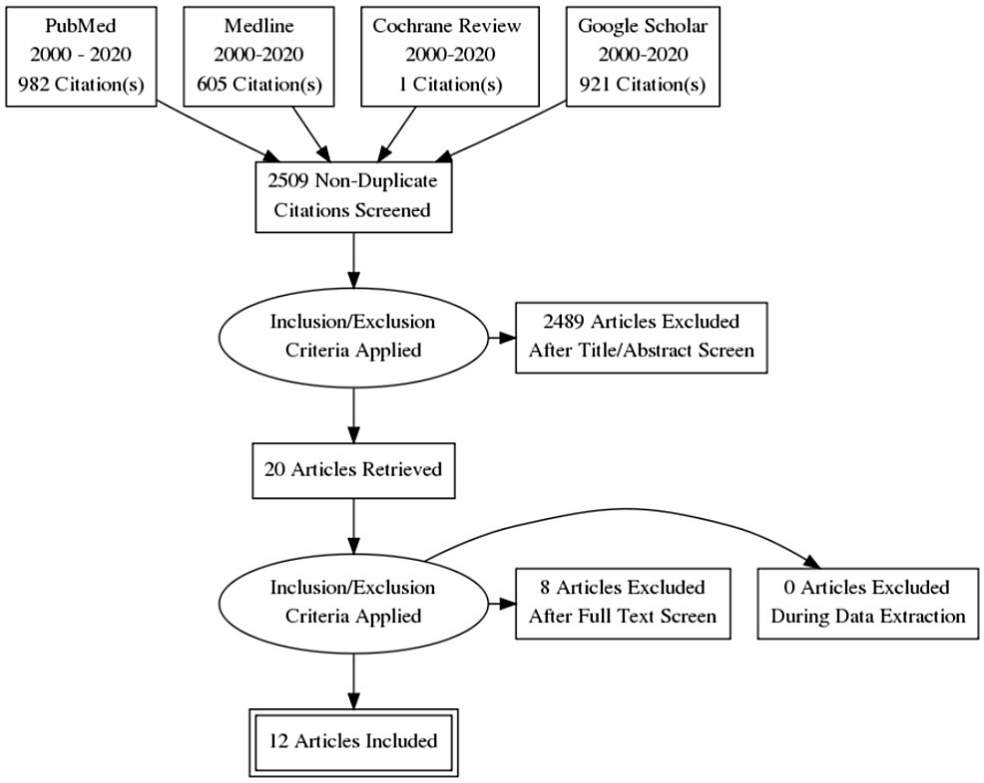
PRISMA Diagram PRISMA: Preferred Reporting Items for Systematic Reviews and Meta-Analyses

A literature review was conducted, to compare post liver resection bleeding in both laparoscopic and open techniques. This search had adhered to the PRISMA (Preferred Reporting Items for Systematic Reviews and Meta-Analyses) (Figure 1). We aimed to identify appropriate comparative studies and clinical trials. The period of review ranged from 2000 to 2020. PubMed, Medline, Cochrane, and Google Scholar were searched to find relevant studies.

Keywords included “laparoscopic hepatectomy”, “Post-operative complications”, “post-hepatectomy bleeding”, “intra-operative bleeding”, “open liver resection”, “hepatocellular carcinoma”, “benign liver tumors”, “minimally invasive”, “hepatobiliary surgery”, “segmentectomy”. The term AND “versus” was used to identify studies that compared the outcomes of both techniques.

Research protocol

Studies comparing the short-term complication of postoperative blood loss in both minimally invasive and traditional open technique liver resection were subject to inclusion and exclusion criteria. The identification, quality assessment, data extraction, and statistical analyses of the studies will be included in the research protocol.

Published papers and studies were searched using the electronic databases described above. The PICO (population, intervention, control, and outcomes) format was also used to help select the most relevant studies that provide a balanced view of the outcomes. Published studies and trials during the period ranging from 2000 to 2020 were included in the review.

Inclusion criteria

All quantitative studies from 2000 to 2020 meeting the following criteria were reviewed in this systematic review: studies and publications highlighting and comparing the postoperative complication of blood loss in LLR and OLR, the indication for each surgery, type of procedure, specific postoperative complications related directly to both techniques, and, most importantly, comparing the degree of blood loss in both techniques. All studies included in this review were published in English and describe a similar patient population.

Exclusion criteria

The exclusion criteria used for this review included the following: no reported outcome allowing for quantifiable comparison, liver resection performed due to metastatic liver disease, studies not published in English, and unable to extract data from results.

Study selection and quality assessment

The Effective Public Health Practice Project (EPHPP) quality assessment tool was used for this review to adequately assess the quality of each publication. It assesses the trustworthiness of each study and ensures the efficiency and reliability of the information included in the publications analyzed. It is a tool that was initially used in the practice of public health but has been proven to be effective in other research fields.

Data extraction and analysis

A data extraction form is utilized to collect the relevant information to address the question set out in this systematic review. The World Health Organization guidelines were also used to assess the complications that can occur after liver resections using a laparoscopic and open technique. Postoperative blood loss is a common complication of surgery and is kept at a minimum as much as possible as bleeding negatively affects postoperative recovery significantly [3].

## Results

Literature search 

A thorough literature search identified 2509 publications using keywords: 982 in PubMed, 605 in Medline, 921 in Google Scholar, and one in Cochrane Reviews (Table 1). Of the 2509 publications, 2489 had been excluded due to non-relevant or non-comparative studies; 20 satisfied the inclusion criteria; 8 were excluded for duplication; and 12 were included in this systematic review. We did not identify any randomized controlled trials (RCTs) that compared the outcomes of LLR and OLR in our literature review. 

**Table 1:** The Search Syntax

PubMed search, Accessed on November 20th, 2020 (921 Articles)	Medline search, Accessed on December 20th, 2020 (605 Articles)	Cochrane Reviews search, Accessed on November 20th, 2020 (1 Article)	Google Scholar search, Accessed on December 20th, 2020 (921 Articles)
(laparoscopic hepatectomy OR Minimally invasive) AND (Post-operative complications OR Post-hepatectomy bleeding OR Intra-operative bleeding) AND (Open liver resection) AND (Versus AND Compare) OR (Hepatobiliary surgery AND Segmentectomy)	(laparoscopic hepatectomy OR Minimally invasive) AND (Post-operative complications OR Post-hepatectomy bleeding OR Intra-operative bleeding) AND (Open liver resection) AND (Versus AND Compare) OR (Hepatobiliary surgery AND Segmentectomy)	(laparoscopic hepatectomy OR Minimally invasive) AND (Post-operative complications OR Post-hepatectomy bleeding OR Intra-operative bleeding) AND (Versus AND Compare)	(laparoscopic hepatectomy OR Minimally invasive) AND (Post-operative complications OR Post-hepatectomy bleeding OR Intra-operative bleeding) AND (Open liver resection) AND (Versus AND Compare) OR (Hepatobiliary surgery AND Segmentectomy)

Demographics 

A total of 12 studies were included in the review, all retrospective in design, which included a total of 287 OLRs and 480 LLRs. The mean blood loss in LLR was 179.1mL and in OLR was 295.64mL. The mean age for OLR was 55.4 years and for LLR was 56.7 years (Table 2) 

**Table 2:** Patient Demographics LLR: laparoscopic liver resection; OLR: open liver resection

Characteristics and demographics	
Studies included in the systematic review	12
Retrospective vs Prospective	All Retrospective
Total Number of Open Resections	287
Total Number of laparoscopic liver resections	480
Mean Blood Loss LLR	179.10ml
Mean Blood Loss OLR	295.64ml
Mean Age (years) in OLR	55.4
Mean Age (years) in LLR	56.7
Mortality (<30 days) in OLR	0
Mortality (<30 days) in LLR	0

Comparing rates of blood loss of LLR and OLR 

The postoperative parameter of blood loss [3] was used to compare the outcomes of LLR and OLR (Table 3). Lesurtel, et al [12], had compared 18 LLR to 20 OLR which had highlighted less blood loss for laparoscopic liver resection at 288mL compared to 485mL for the OLR group. Buell, et al [13] compared the outcome of 17 LLR to 100 OLR for benign tumours. They identified that the mean blood loss for LLR was 299mL compared to 485mL for the OLR group (P0.05). Morino, et al [14] enrolled 60 patients for a comparative study, 30 LLR and 30 OLR. In the LLR group, blood loss was 320mL compared to 479mL in OLR (P≤0.05). Koffron, et al [15] evaluated 300 liver resections, 241 LLR and 59 hybrid cases (OLR and laparoscopic-assisted). They identified that blood loss for LLR was 102mL compared to 325mL loss in the hybrid group. Aldrighetti, et al [16] compared 20 LLR to 20 OLR which showed that blood loss for LLR and OLR was 165mL and 214mL, respectively (P=0.001). Polignano, et al [17] included 50 patients, 25 LLR and 25 OLR, which showed a reduced volume of blood loss in LLR compared to OLR (P≤0.003). Slakey, et al [18] reviewed 62 patients, 45 LLR and 17 OLR, which showed that hospital blood loss was significantly less in the LLR group compared to OLR (P≤0.0001). Qiu, et al [19] compared 49 patients, 24 LLR and 25 OLR, showing a reduced blood loss in the LLR group (210mL in LLR, 380mL in OLR) (P≤0.01). Zhang, et al [1] had evaluated 50 patients (30 LLR to 20 OLR) which indicated reduced blood loss in LLR compared to OLR (200mL vs 328mL) (P<0.05). Nassar, et al [20] also identified reduced blood loss in the LLR group compared to OLR (P≤0.001). However, one study completed by Lau, et al [21] indicated that there was no statistical difference related to blood loss (LLR 386mL, OLR 556mL, P=0.216). Hu, et al [22] also identified no statistical difference when comparing blood loss, 30 OLR and 30 LLR patients were selected for the study (P≥0.05). 

**Table 3:** Selected Studies Comparing the Rates of Blood Loss Of LLR and OLR LLR: laparoscopic liver resection; OLR: open liver resection

Study Author(s)	Year	Location	Study type	Numbers of patients	LLR/OLR	Eligibility for inclusion	Quality assessment	Ethical appraisal
Lesurtel et al	2003	France	Comparative matched	38	18/20	Yes	Yes	Yes
Morino et al	2003	Italy	Comparative matched	60	30/30	Yes	Yes	Yes
Buell et al	2004	USA	Comparative matched	117	17/100	Yes	Yes	Yes
Koffron et al	2007	USA	Comparative matched	300	241/59	Yes	Yes	Yes
Aldrighetti et al	2008	Italy	Comparative matched	40	20/20	Yes	Yes	Yes
Polignano et al	2008	UK	Comparative matched	50	25/25	Yes	Yes	Yes
Hu et al	2011	China	Comparative matched	60	30/30	Yes	Yes	Yes
Slakey et al	2013	USA	Retrospective review	62	45/17	Yes	Yes	Yes
Qiu, et al	2014	USA	Comparative matched	49	24/25	Yes	Yes	Yes
Lau et al	2015	USA	Case matched controlled	125	47/78	Yes	Yes	Yes
Nassar et al	2015	Egypt	Comparative matched	30	15/15	Yes	Yes	Yes
Zhang et al	2015	China	Cohort study	50	30/20	Yes	Yes	Yes

## Discussion

Published reviews on laparoscopic liver resection describe technically challenging cases (peripherally sited tumours, etc.) that may discourage minimally invasive liver resection when compared to the open technique [23]. However, due to the advances in laparoscopic approach over the past three decades, and the added benefit of a perceived reduction in postoperative complications, Koffron, et al [15], have advocated for laparoscopic liver resection to be the gold standard treatment for most hepatectomies. 

Comparing OLR to LLR has been a controversial issue due to the lack of sound evidence, indicating the superiority of LLR to OLR, according to Laurent, et al [24]. Our aim is to compare the postoperative blood loss of both techniques. Till present, there is have been no published randomized controlled trials directly comparing both techniques. Because of the lack of high-quality evidence, it is more difficult to compare both approaches. However, the papers reviewed provide a strong indication that minimally invasive liver resection has reduced intraoperative and postoperative bleeding compared to open liver resection. 

As discussed previously, one study suggested that there is no statistical difference between LLR and OLR in terms of blood loss. This may influence surgeons to opt for open liver resection techniques by comparison to laparoscopic liver resections as per studies by Laurent, et al [24] and Lau, et al [21]. It also reinforces the need for high-quality studies to provide more objective evidence in deciding the appropriate technique to opt for. The technical difficulty in performing laparoscopic liver resection and the demand for further specialized training is another argument against the use of minimally invasive liver resection. Technical issues such as a narrow operative field and the rigidity of minimally invasive instruments may concern surgeons and may have the perception of a higher risk of undesirable complications [23]. Location of tumour sites is another added concern as peripherally sited tumours may prove too complex for resection laparoscopically as seen in the study by Gagner, et al [25].

The study groups that have supported the benefit of LLR comparing OLR in terms of the amount of blood loss also recognize the issue of advanced laparoscopic technique for LLR but advocate the need for continuous development of laparoscopic techniques in light of the results of reduced postoperative complications in general [15].

## Conclusions

Based on the literature available, LLR has a reduced amount of postoperative blood loss, it is an established and safe technique with appropriate training. As discussed previously, higher quality evidence such as randomized controlled trials would provide further insight into the comparative benefits of LLR. This review also only considers the short-term postoperative issue. The blood lost during these studies were measured intra-operatively and the patients’ blood loss post-procedure. In conclusion, this review shows there is a reduced intra-operative blood loss and provides a reasonable argument for the recommendation of LLR for patients undergoing hepatectomies both for benign and malignant conditions. It also provides a good case for the endorsement of further training in laparoscopic techniques related to liver resection, but as discussed, this requires higher quality studies such as RCTs to bolster the argument for LLR.
